# Editorial: The toxicology of drugs of abuse

**DOI:** 10.3389/fpsyt.2023.1142265

**Published:** 2023-01-31

**Authors:** Benjamin M. Ford, Tory R. Spindle, Nicola Simola

**Affiliations:** ^1^Center for Toxicology and Environmental Health North Little Rock, Little Rock, AR, United States; ^2^Department of Psychiatry and Behavioral Sciences, Johns Hopkins University School of Medicine, Baltimore, MD, United States; ^3^Department of Biomedical Sciences, University of Cagliari, Cagliari, Italy

**Keywords:** alcohol, amphetamines, cannabinoids, cathinones, central toxicity, peripheral toxicity, recreational drug use

Several psychoactive substances with abuse potential are used as recreational drugs worldwide, and recreational drug use is a long-standing source of concern for it may lead to addiction as well as other adverse effects. Additional concern on this matter comes from the preclinical and clinical evidence showing that several of the drugs that are used for recreational purposes may have central and peripheral toxic effects of variable severity, some of which may persist even after drug discontinuation (1). The picture of the toxicology of drugs of abuse is further complicated by the continuous appearance of substances belonging to emerging novel chemical classes (i.e., synthetic cannabinoids, synthetic cathinones) that possess unique molecular structure-activity relationships and whose central and peripheral toxic effects are ill defined (2). This Research Topic gathers four articles that present clinical and *in vitro* data on the toxic effects of traditional (i.e., cannabis and ethanol) and emerging (i.e., synthetic cannabinoids and synthetic cathinones) recreational drugs, providing new insights into the toxicology of drugs of abuse.

Smith et al. investigated the effects of smoked cannabis on epigenetic systems, immunological parameters and cannabinoid receptors (CBR) of type 1 and 2 in human peripheral blood cells of regular cannabis users. They found that mRNA for CBR2 increased in blood lymphocytes of cannabis users who had a higher blood concentration of Δ^9^-tetrahydrocannabinol (THC), compared with cannabis users who had a lower THC blood concentration. However, such an increase in the mRNA for CBR2 was not accompanied by changes in immunological markers. Moreover, changes in mRNA for methylation and demethylating enzymes [DNA methyltransferase (DNMT), ten-eleven translocation (TET) methylcytosine dioxygenases] were observed in lymphocytes of cannabis users, suggesting that THC may elicit epigenetic changes. Since increases in DNMT have been linked to some of the pathophysiological processes featuring schizophrenia, the authors suggest that the effects of cannabis observed here should be further investigated in a larger cohort, to ascertain if changes in the levels of DNMT enzymes may be one of the potential mechanisms by means of which cannabis can trigger schizophrenia in vulnerable individuals.

Kazmi et al. performed an exploratory analysis to investigate the existence of associations between pro-inflammatory cytokine markers and subjective measures of sleep quality, scores of anxiety/depression and alcohol consumption among individuals with alcohol use disorder. The results obtained in this study demonstrate that the levels of the cytokines Monocyte Chemoattractant Protein-1 (MCP-1) and interleukin-8 (IL-8) may be associated with higher drinking behavior, poorer sleep quality, and higher anxiety/depression in individuals with alcohol use disorder. Moreover, markers of inflammation and liver function were highly correlated with pro-inflammatory cytokines in individuals with alcohol use disorder, which may suggest a possible relationship between chronic alcohol use and presence of a persistent state of inflammation that may eventually increase the risk of comorbid chronic diseases in individuals with alcohol use disorder.

Albert et al. present a case study of a 21-year-old woman with daily usage of various synthetic cathinones [α-Pyrrolidinopentiophenone (α-PVP, better known as “Flakka”), α-Pyrrolidinohexiophenone (α-PHP), and alpha-Pyrrolidinoisohexaphenone (α-PHiP)] who displayed a severe paranoid psychotic state of mind along with a very pronounced psychomotor restlessness which was diagnosed as akathisia during hospitalization. This case study suggests that use of synthetic cathinones may not only increase the risk of developing psychotic-like sequela, but also increase the risk for development of extrapyramidal symptoms such as akathisia, typically observed during use of amphetamine-like psychostimulants. These findings further remark that it is still very difficult to establish a guideline concerning the treatment of intoxication with synthetic cathinones and dependence thereof.

Kevin et al. selected 7 synthetic cannabinoid receptor full agonists (AMB-FUBINACA, XLR11, PB-22, AKB-48, AB-CHMINICA, CUMYL-PINACA, and 4F-MDMB-BUTINACA), representing several distinct chemotypes and toxicological profiles, and evaluated them in a single-point screen against 241 G protein-coupled receptor targets in antagonist and agonist mode using a cellular β-arrestin recruitment assay. The screening yielded few hits in agonist mode for any compound tested aside from CB1 and CB2 receptors, but many hits in antagonist mode, including a range of chemokine receptors, the oxytocin receptor, and histamine receptors. These findings suggest that some “off-targets” could possibly contribute to the toxidrome of synthetic cannabinoid receptor agonists, particularly at high concentrations, and indicate that CBR1-mediated cellular dysfunction may underlie on-target toxicity of synthetic cannabinoid receptor agonists.

In conclusion, this Research Topic provides up-to-date information that may enhance our understanding of the central and peripheral toxicity elicited by traditional and emerging drugs of abuse. Importantly, better defining the central and peripheral toxicity for traditional and emerging drugs of abuse could offer understanding on how to derive improved interventive therapeutics and regulatory practices for mitigating drug-driven adverse outcomes.

## Author contributions

BF, TS, and NS wrote the editorial and approved it for publication. All authors contributed to the article and approved the submitted version.
